# Long tailed trions in monolayer MoS_2_: Temperature dependent asymmetry and resulting red-shift of trion photoluminescence spectra

**DOI:** 10.1038/s41598-017-14378-w

**Published:** 2017-10-25

**Authors:** Jason W. Christopher, Bennett B. Goldberg, Anna K. Swan

**Affiliations:** 1 Department of Physics, Boston University, 590 Commonwealth Ave, Boston, Massachusetts 02215 USA; 2 Department of Physics and Astronomy, Northwestern University, 2145 Sheridan Road F165, Evanston, IL 60208 USA; 3 Photonics Center, Boston University, 8 St Mary’s St, Boston, Massachusetts 02215 USA; 4 Department of Electrical and Computer Engineering, Boston University, 8 St Mary’s St, Boston, Massachusetts 02215 USA; 5 Searle Center for Advancing Learning and Teaching, Northwestern University, 627 Dartmouth Place, Evanston, IL 60208 USA

## Abstract

Monolayer molybdenum disulfide (MoS_2_) has emerged as a model system for studying many-body physics because the low dimensionality reduces screening leading to tightly bound states stable at room temperature. Further, the many-body states possess a pseudo-spin degree of freedom that corresponds with the two direct-gap valleys of the band structure, which can be optically manipulated. Here we focus on one bound state, the negatively charged trion. Unlike excitons, trions can radiatively decay with non-zero momentum by kicking out an electron, resulting in an asymmetric trion photoluminescence (PL) peak with a long low-energy tail and peak position that differs from the zero momentum trion energy. The asymmetry of the trion PL peak and resulting peak red-shift depends both on the trion size and a temperature-dependent contribution. Ignoring the trion asymmetry will result in over estimating the trion binding energy by nearly 20 meV at room temperature. We analyze the temperature-dependent PL to reveal the effective trion size, consistent with the literature, and the temperature dependence of the band gap and spin-orbit splitting of the valence band. This is the first time the temperature-dependence of the trion PL has been analyzed with such detail in any system.

## Introduction

The two-dimensionality of MoS_2_ reduces dielectric screening, resulting in strong interactions and many-body states such as trions^1^ and bi-excitons^2^. The binding energies of these states in MoS_2_ are nearly an order of magnitude larger than in GaAs quantum wells (QW)^3^, which for trions in MoS_2_ is large enough to make them stable even at room temperature^1^. Like other transition metal dichalcogenides (TMDCs), MoS_2_’s band structure contains two direct-gap inequivalent valleys with identical bands but with opposite spins due to time-reversal symmetry^4^. This symmetry makes it possible to optically address excitations in a specific valley, or coherently generate excitations between valleys^5–7^; effectively endowing the single-particle states with a pseudo-spin degree of freedom called the valley index. Remarkably this pseudo-spin index continues to be conserved in more complicated many-body states. These properties have led to great interest in exciting and manipulating trions to further our understanding of many-body physics and identify unique properties, which may be useful in novel applications such as valleytronics and spintronics.

A trion is formed when either an electron or hole binds to an exciton. In MoSe_2_ and WSe_2_, both positively charged and negatively charged trions have been observed^8,9^, however, only negatively charged trions have been observed in MoS_2_. This difference is due to the high unintentional doping of MoS_2_ that typically leaves samples with an electron density near 10^13^ cm^−2^ ^1^; too large to be completely neutralized via electronic back-gating on SiO_2_/Si^++^ substrates. These excess electrons greatly increase the likelihood that an exciton and electron meet and bind into a trion, which gives rise to the large trion population observed in photoexcited MoS_2_.

Many properties of trions in MoS_2_ are already established. Valley selectivity and binding energy were investigated in the first reported observation of trions^1^. Several experiments have monitored trion population while electrically^1^ or chemically doping^10^. Recent ultra-fast THz transmission measurements of MoS_2_ found that the larger mass of the trion relative to the electron results in negative photo-conductivity^11^. Importantly, the negative photo-conductivity enabled C. H. Lui *et al*.^11^ to separate the trion and electron contributions to the photo-conductivity, providing direct evidence of trion transport^11^. These early observations show that trions have pseudo-spin and charge, and are abundant when MoS_2_ is photoexcited. These properties make trions interesting candidates for both scientific and technological pursuits because trion transport, density, and pseudo-spin can be easily controlled with electric fields and polarization.

**Figure 1:**
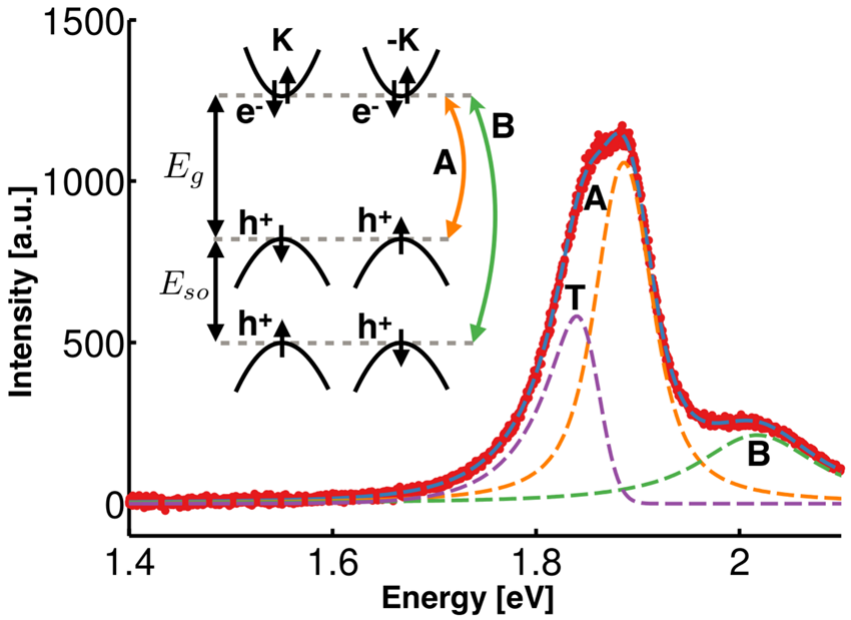
PL spectrum of MoS_2_ at 273 K (background subtracted). Raw spectra are included in Supplementary Information S1. A and B denote excitons, and T denotes the trion. Note the trion asymmetric shape with a characteristic long low-energy tail. Inset: Band structure near the K and −K points of the Brillouin zone with band gap, *E*_*g*_, ~1.9 eV, and spin-orbit splitting, *E*_*so*_, ~140 meV.

In this article, we probe the thermal distribution and effective size of trions by measuring the long low-energy tail of the trion PL spectra over a wide range of temperatures. During radiative decay, an electron or hole is kicked out of the trion, carrying away appreciable momentum, leaving the net zero-momentum electron-hole pair to recombine. This allows non-zero momentum trions to radiatively decay and is responsible for the long, low-energy tail of the trion PL spectrum. In Fig. 1 an example of such a long tail can be seen in the trion component of the fit to the spectra, and is in contrast with the two symmetric peaks corresponding with the A and B exciton fit components. As indicated in the inset, the A and B excitons emerge from spin-orbit splitting of the valence band. As will be discussed in detail below, the trion low-energy tail length depends on two factors: the trion thermal momentum distribution, and the bound state wave function. We vary temperature to change the momentum distribution in order to explore these two factors enabling estimation of the trion size.

To the best of our knowledge, the temperature dependence of the trion tail length has not been analyzed in detail for any system. The most extensive work prior to ours observed the trion tail in MoSe_2_ only for temperatures below 70 K^8^, which is where the tail is shortest and the peak almost symmetric. Further, Ross *et al*.^8^ did not analyze the temperature dependence of the trion tail length to separate the thermal and wave function components. We also show for the first time that the contribution to the PL from non-zero momentum trions leads to a temperature dependent PL peak offset between the zero momentum trion energy, ${E}_{tr}^{0}$, and the energy with highest trion PL intensity. As temperature increases and more non-zero momentum trions contribute to the PL, the trion peak becomes increasingly asymmetric increasing the peak offset from ${E}_{tr}^{0}$. Our analysis allows us to correct for peak shifts caused by asymmetry and is equally applicable to trions in any system.

## Results

### Trion Photoluminescence Theory

**Figure 2:**
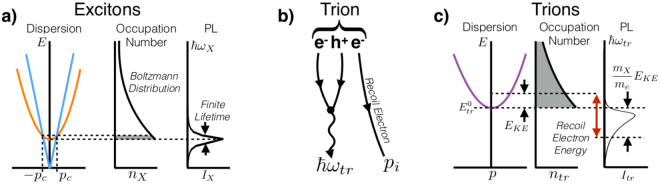
(**a**) The exciton dispersion is shown in orange and photon dispersion in blue with exaggerated momentum, ~200×, to make the light cone visible. Only excitons within the light cone, |*p*|<*p*_*c*_, can radiatively decay. This population is highlighted in the narrow, delta function like region in the occupation number plot, which results in a Lorentzian shaped PL. (**b**) The Feynman diagram for trion radiative decay. One of the electrons recombines with the hole to emit a photon while the other electron is kicked out to conserve energy and momentum. (**c**) The trion dispersion is shown in purple with the zero-momentum and kinetic energies of a trion denoted. All trion states can radiatively decay, so all states in the occupation plot are allowed optical transitions. To convert from the occupation distribution to the PL, we must account for the energy of the recoil electron resulting in the long low-energy tail.

The trion spectral shape is heavily influenced by the momentum distribution of trions, and the momentum dependent trion decay rate. To gain intuition for the trion spectral shape we momentarily disregard the momentum dependence of the trion decay rate. When an exciton decays, all of its momenta must be carried away by the emitted photon, so only excitons within the light cone, |*p*| < *p*_*c*_, can radiatively decay, see Fig. 2a. This small population of excitons appears like a delta function in occupation space, which when convolved with a Lorentzian to account for the phenomenological finite lifetime creates the exciton Lorentzian PL peak. Trions, on the other hand, eject an electron when they radiatively decay as shown in Fig. 2b. The recoil electron carries away all of the trion’s momentum, allowing all trions to decay radiatively. The corresponding PL spectrum resulting from a Boltzmann distribution of trion kinetic energies is shown in Fig. 2c. Our use of the Boltzmann distribution is well supported by thermalization time and lifetime measurements, details of which are included in the Supplementary Information S2. Including the energy of the recoil electron is essential when determining the PL spectral shape from the trion momentum distribution. The emitted photon energy is given by $\hslash {\omega }_{tr}={E}_{tr}^{0}-\frac{{m}_{X}}{{m}_{e}}{E}_{KE}$, where ${E}_{tr}^{0}$ is the zero-momentum trion energy, *m*_*X*_ is the exciton mass, *m*_*e*_ is the effective electron mass, and *E*_*KE*_ is the trion kinetic energy. Thus the PL spectrum is the thermal trion population distribution “flipped” over the zero-momentum trion energy and magnified by the ratio *m*_*X*_/*m*_*e*_ as shown in Fig. 2c. The flipping means that the higher energy, non-zero momentum trion states create a *low* energy tail containing information about the thermal distribution and effective electron and hole masses.

To accurately analyze the trion PL shape we need to account for the fact that trions with different momenta will decay at different rates. This effect is accounted for in the optical matrix element, *M*(*p*), which is a function of the trion momentum, *p*. Based on theory as well as experimental observations in GaAs quantum wells and TMDCs, it is known that the optical matrix element is well approximated by an exponential of the trion kinetic energy^3,8,12^. In the limit that the trion wave function takes the form of a Gaussian wave packet, this approximation becomes exact (see Supplementary Information S3). With that insight in mind, we approximate the optical matrix element, *M*(*p*), as $M(p)\propto \exp [-{(\frac{{m}_{X}}{{m}_{tr}}\frac{a}{\hslash })}^{2}{p}^{2}]$ where *a* is the standard deviation of the Gaussian wave packet, which we interpret as the effective size of a trion. Details of the optical matrix element calculation are included in the Supplementary Information S3.

Accounting for the optical matrix element and the thermal distribution of momenta, the trion PL intensity is given by

1a $${I}_{tr}(\hslash \omega )=\exp [-({E}_{tr}^{0}-\hslash \omega )/\varepsilon ]{\rm{\Theta }}({E}_{tr}^{0}-\hslash \omega )/\varepsilon $$

1b $$\frac{1}{\varepsilon }=\frac{{m}_{e}}{{m}_{X}}\frac{1}{{k}_{B}T}+\frac{{m}_{X}}{{m}_{tr}}\frac{4{m}_{e}{a}^{2}}{{\hslash }^{2}}$$

where Θ is the unit step function, *T* is temperature, *k*_*B*_ is Boltzmann’s constant, and *ε* is the length, in units of energy, of the low-energy tail of the trion PL. The spectrum described by equation (1a) is normalized to have a total integrated area of 1, which does not take into account thermal disassociation of trions at elevated temperatures. Nor does equation (1a) include phenomenological broadening, which we include by convolving equation (1a) with a Lorentzian. The first term in equation (1b) for 1/*ε* comes from the Boltzmann distribution of trion momenta, is temperature dependent, and is a function of the hole to electron mass ratio. The second term comes from the optical matrix element, is temperature independent, and is a function of the trion size. At low temperatures, the Boltzmann term will dominate and the tail length will be small creating a nearly symmetric peak shape. As the temperature is increased, the temperature independent term will dominate, and the energy tail length will increase until it saturates at a value dictated by the size of the trion. By measuring the tail length at different temperatures, we can separate these two terms, and determine the hole to electron mass ratio and the effective trion size multiplied by the electron mass.

As illustrated in Fig. 2c the peak in the PL intensity is red-shifted relative to the zero-momentum trion position. The convolution of equation (1a) with a Lorentzian rounds the sharp corner caused by the step function, and red-shifts the energy with highest intensity by an amount that depends on the width of the Lorentzian and the trion tail length. The longer the tail, the larger the red-shift. And the wider the Lorentzian, or shorter the trion lifetime, the larger the red-shift. As temperature increases the tail length gets longer and the Lorentzian gets wider, so the red-shift increases as well. The red-shift in trion PL peak position with temperature has been observed before in GaAs quantum wells^13^ but without analysis. We note that absorption spectroscopy will also have a red-shifted trion peak position, but with a reduced magnitude (see Supplementary Information S4).

### Experiment

To study the temperature dependence of the trion tail length, we prepared monolayer MoS_2_ flakes via standard mechanical exfoliation onto silicon wafers with 300 nm of thermal oxide. After exfoliation, monolayer samples were identified optically and verified via PL and Raman spectroscopies. Spectra were obtained at temperatures ranging from 83 K to 473 K in steps of 25 K. We stayed above liquid nitrogen temperatures because theory suggests that at temperatures lower than ~70 K the tail length is very small and difficult to extract with any accuracy. Further, we also found it difficult to extract the trion tail length at temperatures above ~300 K because the trion peak becomes weaker due to thermal disassociation, and the thermal broadening of the peak dwarfs the tail length. For this reason, we focused our efforts on measurements made between 83 and 300 K.

#### Band Gap and Spin-Orbit Coupling Temperature Dependence

**Figure 3:**
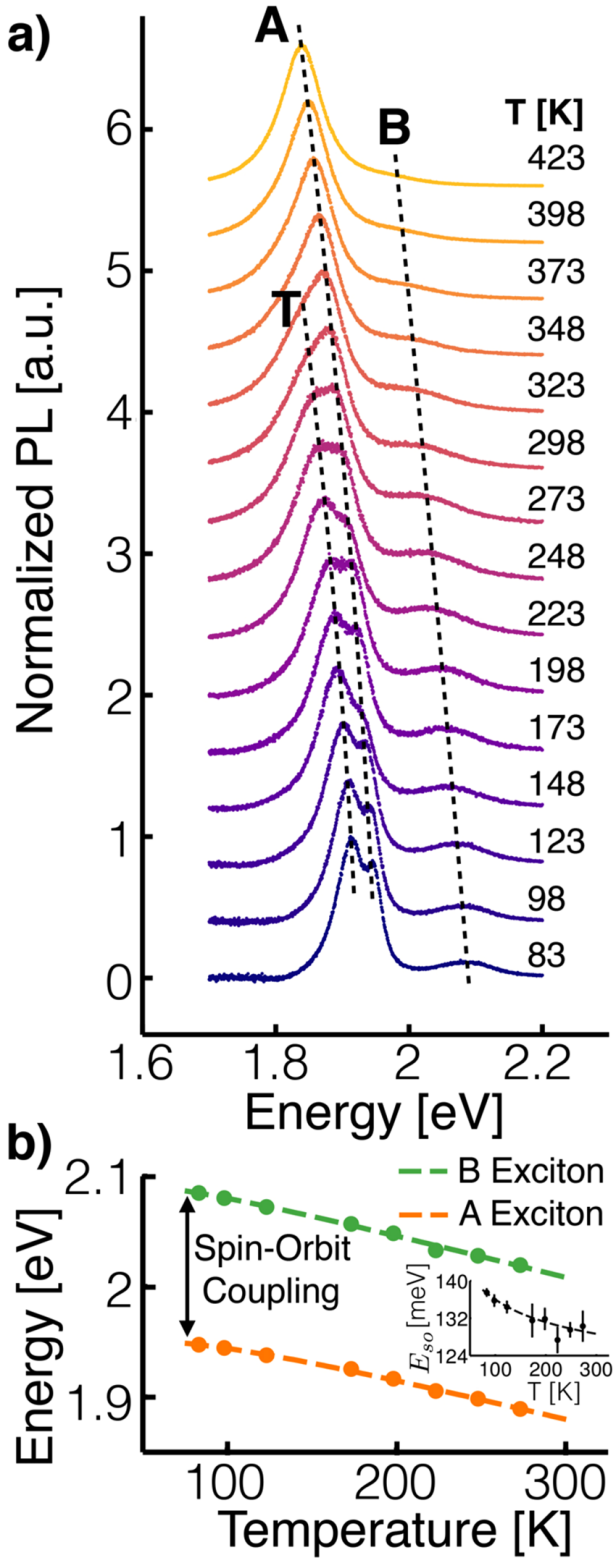
(**a)** Monolayer MoS_2_ PL spectra measured from 83 K to 423 K. The background has been removed from the spectra, and the spectra have been normalized to make features easily visible. Guides to the eye for the trion, A exciton and B exciton peak positions are included for clarity. (**b)** A exciton and B exciton peak positions versus temperature and best-fit to a semi-empirical model^14,15^. For most data points the error bars are smaller than the symbols. Inset: Temperature dependent spin-orbit splitting between A and B exciton energies.

The measured spectra shown in Fig. 3a are in good agreement with our qualitative expectations. First, note that at and below 123 K the trion and A exciton peaks are clearly distinct, indicating high sample quality and little broadening due to impurities. At higher temperatures, thermal excitations reduce the populations of bound states and shorten the trion and exciton lifetimes resulting in a less prominent, broader trion peak until it completely disappears at ~348 K. Additionally, we see that both the exciton and trion peaks red-shift as temperature increases, because of thermal expansion^14,15^. We find the temperature dependence of the A and B excitons to be well described by a semi-empirical model based on electron-phonon coupling^15^, see Fig. 2b. In this model, the exciton energy is

2 $${E}_{X}(T)={E}_{X}^{0}-S\langle \hslash \omega \rangle [\coth (\langle \hslash \omega \rangle \mathrm{/2}{k}_{B}T)-1]$$

where ${E}_{X}^{0}$ denotes the zero temperature exciton energy, $\langle \hslash \omega \rangle$ represents the average phonon energy contributing to the temperature change of the exciton energy, and *S* is the effective electron-phonon coupling constant. Best-fit values for ${E}_{X}^{0}$, $\langle \hslash \omega \rangle$, and *S* for the A and B excitons are shown in Table 1 along with results from measurements done before the trion was discovered^14^. There is good agreement with the previous measurements with the exception of the zero temperature energy. Given that the trion was not known at the time of the earlier work, we suspect that the low-temperature spectra were fit to the trion peak instead of the A exciton peak, which would account for the significant difference in zero temperature energy. Recalling that the A and B excitons are split by spin-orbit coupling, and noting that $\langle \hslash \omega \rangle$ and *S* are different for the A and B exciton, we conclude that the spin-orbit splitting of the valence band slightly decreases as temperature increases.

**Table 1:** Table of parameter values describing the exciton energy temperature dependence using equation (2).

	${{\boldsymbol{E}}}_{{\boldsymbol{X}}}^{{\bf{0}}}$ [eV]	${\boldsymbol{\langle }}{\boldsymbol{\hslash }}{\boldsymbol{\omega }}{\boldsymbol{\rangle }}$ [meV]	*S* [−]
A	1.952 ± 0.003	23 ± 6	2.2 ± 0.3
B	2.094 ± 0.005	16 ± 6	2.3 ± 0.2
A^14^	1.86	22.5	1.82

The bottom row shows previous measurements done without accounting for the trion contribution to the PL^14^.

#### Trion Tail Length and Zero-Momentum Trion Energy

**Figure 4:**
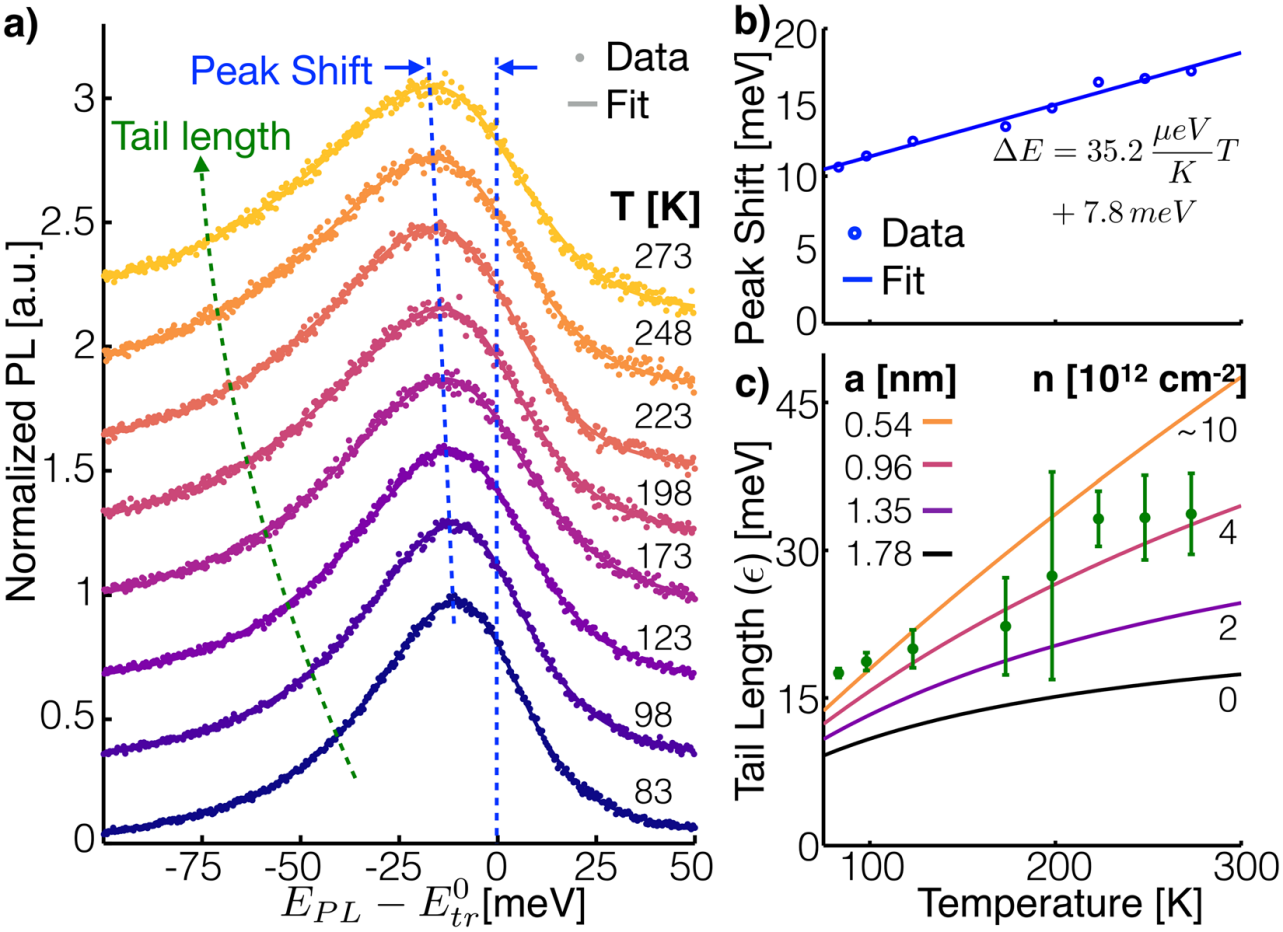
(**a**) Trion contribution to the PL. The data points are the PL with background and exciton contributions subtracted, and the fit is only the trion portion of the fit function. As the temperature increases, the peak position red-shifts and the low-energy tail gets longer. (**b**) Difference between the energy with highest intensity and ${E}_{tr}^{0}$ from the spectra in panel a. (**c**) Extracted trion tail length, *ε*, as well as theoretical curves assuming various values for *a*.

Figure 4 contains three panels to highlight the changes to the trion PL spectrum with temperature. Panel a shows the trion fit component of the spectra at each temperature, and data with contributions due to background and excitons subtracted. Dashed lines, as guides for the eye, are drawn to show how peak shift and tail length evolve with temperature, and panels b and c further focus on each of these important features. Qualitatively, we see the expected temperature dependent effects to the trion spectra. As predicted by our theory developed above, the low-energy tail gets longer and the peak red-shifts relative to the zero-momentum trion energy as the temperature increases. Note that this red-shift is in addition to the red-shift caused by band gap renormalization discussed in the previous section.

To highlight the magnitude and temperature dependence of the red-shift we plot the difference between the energy with the highest PL intensity and ${E}_{tr}^{0}$ in Fig. 4b as calculated from the curves in Fig. 4a. This represents the error that would be made in fitting the trion peak with a Lorentzian and ascribing the zero-momentum trion energy to the peak position. In such a case, ${E}_{tr}^{0}$ would be measured incorrectly by nearly 20 meV at room temperature. The temperature dependence of the red-shift relies on trion properties in a complicated form (see Supplementary Information S6 for details) making it difficult to gain further physical insight from this data without considerable speculation. However, over the temperature range studied, the shift is fit well by a linear function, which we have included along with fit parameters in the Fig. 4b. The fit provides a simple means of comparison with future experiments, and a method for others to correct for the peak shift in analysis done using a Lorentzian fit function.

Figure 4c shows our extracted trion tail lengths, which increase with temperature as expected. Fitting the tail lengths to equation (1b), the best-fit value for the effective trion size, *a*, is 0.54 nm assuming quasiparticle self-consistent *GW* determined values for the effective electron and hole masses^16^. For comparison, we have included in Fig. 4c tail length versus temperature curves for several different values of *a*. The topmost curve is the best-fit line to the data. Two important references to compare our result with are a measurement made using absorption spectroscopy^17^ and a theoretical prediction^18^. Both of these references used more complicated trion wave packets in their analysis than we have, so in order to make a fair comparison we derived values of *a* for these references by minimizing the difference between the optical matrix elements of the Gaussian wave packet we use and the more complicated wave packets used in the references (see Supplementary Information S3 for details). The curves in Fig. 4c with *a* equal to 0.96 and 1.35 nm sizes correspond with the derived values for *a* from the absorption spectroscopy reference^17^ with two different levels of electron doping. The curve with *a* = 1.78 nm corresponds with the derived value from the theoretical prediction^18^. The relative position of the curves is explained by the high temperature behavior of equation (1b), which shows that the tail length saturates at a value proportional to *a* ^−2^, placing curves for larger values of *a* below those for smaller values. However, a low temperature expansion of equation (1b) reveals that the lowest order term is proportional to temperature and independent of *a* (see Supplementary Information S7 for details), forcing the curves to merge at low temperature explaining the tighter spacing between curves at lower temperature. Given the tighter spacing at lower temperatures, our lower temperature data is less sensitive to the value of *a*, and leads us to suspect that our two lowest temperature data points skew the best-fit value for *a* too low. This possibility is qualitatively supported by the good agreement of our data, excluding the two lowest temperature points, with the 0.96 nm curve.

## Discussion

An alternative explanation for the low-energy trion tail is the existence of additional peaks such as trapped states or a splitting between inter- and intra-valley trions^19^. While these possibilities cannot be ruled out completely without further measurements, it is unlikely that additional peaks would imitate the observed low-energy tail, especially the temperature dependence. There are two characteristics of trapped state peaks in MoS_2_ that allow us to rule them out. First, at the low end of the temperature range of our measurements, trapped state peaks are observed as isolated, low energy peaks^20–22^ that could not be confused with a low-energy tail on the trion peak. Second, the temperature dependence of trapped state peak amplitudes is opposite of what is necessary to explain the observed trion tail length temperature dependence. As temperature increases trapped state peaks decrease in amplitude^20–22^. If a trapped state peak was responsible for a low-energy shoulder on the trion peak, then as temperature increased the shoulder would decrease in size, producing a tail length that decreased as temperature increased, contrary to our observations. In support of the characteristics of trapped state peaks reported in the literature, we too observe an isolated, low-energy peak at low temperatures (see Supplementary Information S1). Our observation of a trapped state peak provides additional assurance that the low-energy tail is not due to a trapped state peak. As for inter- and intra-valley trions, the higher energy inter-valley trion decays rapidly, ~1 ps^19^, into the lower energy intra-valley trion. Since the transition rate from inter- to intra- is large relative to the optical decay rate^23^, the vast majority of PL comes from the intra-valley trion, and the inter-valley trion, if anything, would create a high-energy tail in contrast to the low-energy tail we observe. For these reasons we are confident that the long tail is due to the decay of non-zero momentum trions and not other peaks.

Substrate dielectric and electron density can greatly affect the trion binding energy and effective tion size because they alter charge screening. The substrate dielectric will change the external screening, screening that occurs outside of the monolayer, while the electron density changes the internal screening, screening that occurs within the monolayer. Keeping these two forms of screening in mind, we return to the references we highlighted in the previous section regarding trion size. The theoretical prediction^18^ assumed vacuum as the substrate and zero electron density, neither of which match our experiment or that of the absorption spectroscopy measurements. This gives one explanation for why the theoretical prediction differs from the experimental measurements. The absorption spectroscopy measurements^17^ were performed on a quartz substrate which has a nearly identical dielectric constant as our SiO_2_ substrate, so we expect the external screening to be similar. Additionally, Zhang *et al*.^17^ did substantial work to calculate the trion radius accounting for external screening and internal screening as doping changes. They found that as doping *increases* from 2 × 10^12^ cm^−2^ to 4 × 10^12^, the trion size *decreased* from 1.35 nm to 0.96 nm. We have incorporated this information into Fig. 4 by labeling the curves with electron density on the right edge, which shows a monotonic trend from 0 electron density, the theoretical calculation, up to ~10 × 10^12^ cm^−2^, a likely estimate of the electron density in our sample given observations in similar samples^1^. This trend suggests that heavy electron doping offers an explanation for the smaller trion radius found in our experiment.

This trend is counter to standard intuition that *increased* doping strengthens screening, and, based on the hydrogen atom, strengthened screening leads to an *increased* radius. However, in the 3D hydrogen model there is only one form of screening unlike the external and internal components of 2D materials. With suitable scaling of the spatial coordinates and eigenvalues, the hydrogen model can be nondimensionalized. This makes it easy to determine how screening changes the binding energy and wave function; they are simply rescaled appropriately. To account for the internal and external screening in a 2D system embedded in a 3D environment, the proper interaction potential introduces a new length scale^24,25^ which makes it impossible to nondimensionalize the 2D exciton Schrödinger equation, and it is no longer as simple as rescaling to determine the effect of screening on binding energy or wave function. Hence, we cannot be certain of how screening will change the trion radius without resorting to numerics like Zhang *et al*.^17^, and intuition from the hydrogen atom no longer applies.

In our analysis we assumed that the trion wave function is temperature independent, but this is only approximately true. As the temperature changes, the band-gap renormalizes altering the dielectric function which, as discussed above, changes the trion wave function. This offers an explanation for why our data do not follow the theoretical curves in Fig. 4c exactly.

## Conclusion

In this article, we have discussed the physical mechanism through which non-zero momentum trions radiatively decay and how that results in an asymmetric PL peak with a long, low-energy tail. By accounting for the long tail in our fits to spectra measured over a wide range of temperatures, we were able to estimate the trion effective size, determine the temperature dependence of the A and B exciton energies, and resolve the temperature dependent spin-orbit coupling for the first time. We find that our trion size is consistent with doped MoS_2_ as measured using absorption spectroscopy. We have further shown that the zero momentum trion energy, ${E}_{tr}^{0}$, will be erroneously determined when using a symmetric, Lorentzian peak, which will result in over-estimating the trion binding energy.

The analysis we have presented can be used to study trions in other systems such as MoSe_2_ and WSe_2_ and applied to heterostructures of TMDCs where only the interlayer excitons^26^ have been investigated. Experiments on MoSe_2_ or WSe_2_ would require back-gating due to the small unintentional doping from exfoliation in order to have a sufficient number of trions for good signal to noise. However, adding the back-gate will need to be done with great care to preserve sample quality. In the case of interlayer trions, our method could add additional benefit by estimating the hole to electron mass ratio. While not pursued here, it is clear that the first term of 1b contains precisely this information. For interlayer trions, this would be crucial information in determining which layer contains the electrons and which contains holes.

The red-shift of the trion peak and the long low-energy tail could be used to understand the effect of doping on the dielectric function. The complicated relationship between charge and screening is still not well understood in TMDCs. However, theory can make direct predictions for the trion size and binding energy, which can be compared with experiments using the methods in this article. It is typically difficult to extract this information from excitons since it requires identifying excited exciton states which have a small oscillator strength^27^. This suggests that a systematic way to probe the doping effect on the dielectric function in TMDCs is to back-gate a sample and measure the trion binding energy and tail length as a function of gate voltage. Such an experiment will still require more elaborate theoretical work to properly interpret the indirect observations.

## Methods

### Sample Preparation

Bulk MoS_2_ crystals were obtained from SPI Supplies, and mechanically exfoliated down to few-layers using low adhesion cleanroom tape (UltraTape). Clean substrates of degenerately doped silicon with 300 nm of thermal oxide, ideal for optical contrast^28,29^, were prepared via isopropyl alcohol and acetone sonication baths for 10 minutes each, followed by piranha etch for 20 minutes to remove all traces of contamination.

### Photoluminescence Spectroscopy

We controlled sample temperature using a Linkham THMS 600 cryostat, and excited our samples with a 514.5 nm Argon ion laser at 250 *μ*W to avoid heating the sample. The laser was focused to a spot size of ~790 nm using an extra long working distance Mitutoyo 100x objective (0.7 NA). Spectra were obtained using a Renishaw spectrometer with a 600 lines per mm grating. Details of our fitting method and statistical analysis can be found in the Supplementary Information S5.

### Data Availability

The datasets generated during and/or analyzed during the current study are available from the corresponding author on reasonable request.

## Electronic supplementary material

Supplementary Information
