# Supplementary material for: Long tailed trions in monolayer MoS2: Temperature dependent asymmetry and resulting red-shift of trion photoluminescence spectra
Source: Sci Rep. 2017 Oct 25;7:14062. doi: 10.1038/s41598-017-14378-w (PMC5656673; doi:10.1038/s41598-017-14378-w)
Supplement: Supplementary file 1 — Supplementary Information [file 41598_2017_14378_MOESM1_ESM.pdf]

Supplementary Information for Long tailed trions in monolayer MoS_2: Temperature dependent asymmetry and resulting red-shift of trion photoluminescence spectra

Jason W Christopher[1], **Bennett B Goldberg**[1,2,5], **and Anna K Swan**[1,3,4,*]

[1]Department of Physics, Boston University, 590 Commonwealth Ave, Boston, Massachusetts 02215, USA
[2]Department of Physics, Northwestern University, 2145 Sheridan Road F165, Evanston, IL 60208, USA
[3]Photonics Center, Boston University, 8 St Mary's St, Boston, Massachusetts 02215, USA
[4]Department of Electrical and Computer Engineering, Boston University, 8 St Mary's St, Boston, Massachusetts 02215, USA
[5]Searle Center for Advancing Learning and Teaching, Northwestern University, 627 Dartmouth Place, Evanston, IL 60208, USA
[*]swan@bu.edu

S1 Raw PL Spectra

The raw spectra used in our analysis are shown in Fig. S1 with the trapped exciton peak at low temperatures clearly noted.

S2 Trion Thermal Distribution

Ultra-fast pump-probe experiments find the carrier thermalization time in MoS_2 to be ∼20 fs[1], while THz pump-probe spectroscopy shows the combined trion non-radiative and radiative lifetime to be ∼30 ps[2]. Since these two time scales differ by three orders of magnitude, our assumption of thermal equilibrium is well founded. Further, estimates of trion density based on laser power, spot size and quasi-particle lifetimes show that in our experiment we will not achieve trion densities sufficient for quantum degeneracies, supporting our use of the Maxwell-Boltzmann distribution for trion momentum.

S3 Optical Matrix Element Calculations and Adaptations

The optical matrix element, $M(\mathbf{p})$, which describes the momentum dependent probability of radiative decay of a trion is given by[3]

$$M(\mathbf{p}) \propto \int \mathrm{d}^2\rho\, \psi_{tr}(\boldsymbol{\rho}_1 = \mathbf{0}, \boldsymbol{\rho}_2 = \boldsymbol{\rho}) \exp\left(-i\frac{\mathbf{p}\cdot\boldsymbol{\rho}}{\hbar}\frac{m_X}{m_{tr}}\right). \tag{S1}$$

where ψ_{tr} is the trion wave function, and $\boldsymbol{\rho}_1$ and $\boldsymbol{\rho}_2$ are the locations of the electrons relative to the hole. The matrix element is computed with one of the electrons having a relative coordinate of zero, which makes intuitive sense as one of the electrons should be recombining with the hole at the origin. Hence, the optical matrix element is just the Fourier transform of the trion wave function's second electron position with the first electron position set to zero. If we make the substitution into equation (S1) that the trion wave function is a Gaussian packet with standard deviation a, $\psi_{tr}^{1\mathrm{P}}(\mathbf{0},\boldsymbol{\rho}) \propto \exp\left(\frac{-\rho^2}{4a^2}\right)$, then $M^{1\mathrm{P}}(\mathbf{p}) \propto \exp\left[-\left(\frac{m_X}{m_{tr}}\frac{a}{\hbar}\right)^2 \mathbf{p}^2\right]$ and we can interpret a as the effective trion size as discussed in the text.

We have emphasized in the previous paragraph that the Gaussian wave packet corresponds with a one parameter trion wave function by using the superscript 1P, the single parameter being the effective trion size, a. However, the absorption spectroscopy work[4] and theoretical calculations[5] both used more realistic two parameter trion wave functions as follows. It is expected, based on observations in GaAs quantum wells, that the two electrons will form a singlet state, in which case ψ_{tr} must be symmetric when swapping the positions of the two electrons. A standard way to incorporate this symmetry is to form the symmetric product of two single particle wave functions. In the absorption spectroscopy work[4] and theoretical calculations[5] the

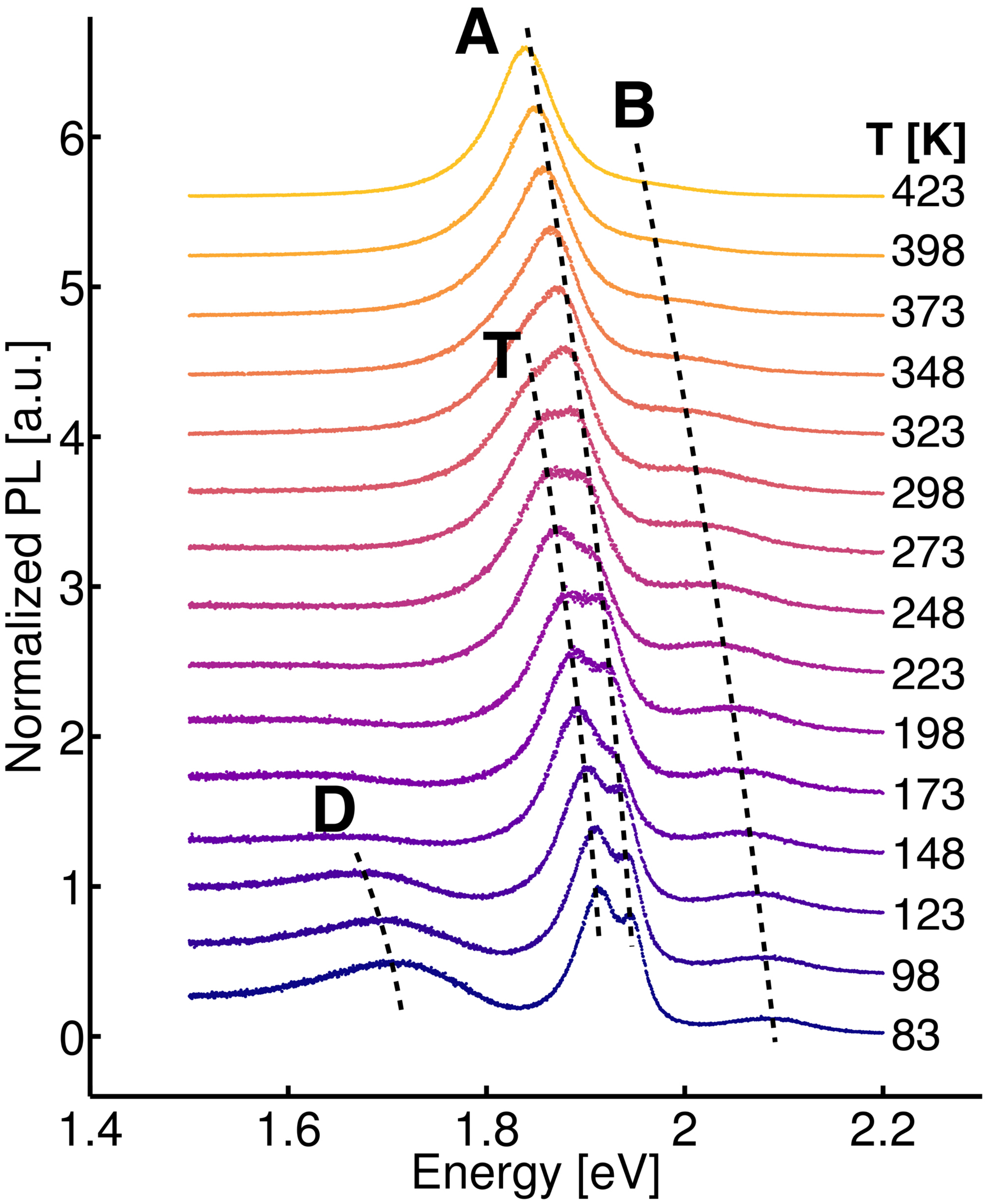

Figure S1. Waterfall plot of data before removing the trapped exciton peak, marked with **D**, and an exponential tail from lower energy defect states.

author	Substrate	n_e [cm^{-2}]	b [nm]	c [nm]	⇒	a [nm]
Zhang *et. al.*[4]	Quartz	4×10^{12}	0.83	1.08	⇒	0.96
Zhang *et. al.*[4]	Quartz	2×10^{12}	0.93	1.77	⇒	1.35
Berkelbach *et. al.*[5]	Vacuum	0	1.03	2.52	⇒	1.78

Table S1. Table of two parameter, b and c, wave function sizes along with adapted single parameter wave function size, a.

single particle wave function is chosen to be the zero angular momentum 2D hydrogen atom wave function

$$\psi_X(\boldsymbol{\rho};a) = \sqrt{\frac{2}{\pi a^2}} e^{-\rho/a} \tag{S2}$$

where the X denotes exciton since this is expected to be the lowest energy exciton wave function, and a is a free parameter for the size of the orbital. The trion singlet state wave function formed from this single particle wave function is

$$\psi_T^{2\mathrm{P}}(\boldsymbol{\rho}_1,\boldsymbol{\rho}_2;b,c) \propto \psi_X(\boldsymbol{\rho}_1;b)\,\psi_X(\boldsymbol{\rho}_2;c) + \psi_X(\boldsymbol{\rho}_2;b)\,\psi_X(\boldsymbol{\rho}_1;c) \tag{S3}$$

where the superscript 2P denotes that this is a two parameter wave function with parameters b and c that describe the size of the two electron orbits about the hole.

To compare results from Berkelbach *et. al.*[5] and Zhang *et. al.*[4] with ours we have adapted their values for b and c by finding the value of a that minimizes the sum square error (SSE) between the optical matrix elements of their two parameter wave function and our single parameter wave function. The SSE is given by

$$\mathrm{SSE} = \int \mathrm{d}^2 p \left[M^{1\mathrm{P}}(\mathbf{p}) - M^{2\mathrm{P}}(\mathbf{p})\right]^2 \tag{S4}$$

where $M^{1\mathrm{P}}$ is the matrix element given by our one parameter, Gaussian wave function, and $M^{2\mathrm{P}}$ is the matrix element given by the more complicated two parameter, symmetrized hydrogen atom wave function. The values of a that minimized the SSE for different values of b and c found in the literature are shown in Table S1.

S4 Absorption Spectroscopy Trion Peak Shift

As discussed in the main text, the trion peak is red-shifted relative to zero momentum trion energy because of the convolution of the phenomenological broadening with the asymmetric intrinsic spectrum. The Feynman diagram for trion decay can be run in the reverse time direction to create the diagram for optical absorption via trion formation, so the absorption spectra will also have a low energy tail just like equation (1a). However, because the absorption will be proportional to the occupation number of electrons (which are being captured by the photo excited excitons to create trions) equation (1b) will be modified slightly to

$$\frac{1}{\varepsilon} = \frac{m_{tr}}{m_X}\frac{1}{k_B T} + \left(\frac{m_X}{m_{tr}}\right)^2 \frac{4 m_e a^2}{\hbar^2}\,(m_e \to m_{tr} \text{ in the first term}), \tag{S5}$$

which makes the temperature dependent first term roughly 3 times larger than in the PL case. This means that the absorption tail length at temperature T is approximately equal to the PL tail length at temperature $T/3$. The peak red-shift is determined by the trion tail length and lifetime, so if we neglect thermal changes to the trion lifetime, then Fig. 4b & c can be used as a mapping from tail length to peak red-shift. Under this approximation, the red-shift of the absorption peak at temperature T is simply the red-shift of the PL peak at temperature $T/3$. Since the red-shift monotonically increases with temperature, the peak shift observed in absorption spectroscopy will always be smaller than the shift observed in PL spectroscopy. For example, at room temperature the PL peak is red-shifted by $\sim$15 meV, but the absorption peak is expected to red-shifted by only $\sim$10 meV (the red-shift in Fig. 4b at 100 K). In short, the red-shift of the absorption trion peak will be less than the PL peak.

S5 Fitting Method and Statistical Analysis

We fit our measured spectra to two Lorentzian peaks for the A and B excitons, and a long-tailed peak for the trion using equation (1a) convolved with a Lorentzian to account for the finite trion lifetime. Additionally, we accounted for inhomogeneous broadening[6–8] by convolving the fit function with a Gaussian. This broadening is relatively small at the lower temperatures where it is less than half the FWHM of any of the peaks, but is increasingly important at the higher temperatures where it becomes the same size as the Trion FWHM, the smallest FWHM of any of the peaks. In total, these peaks amount to 11 fit

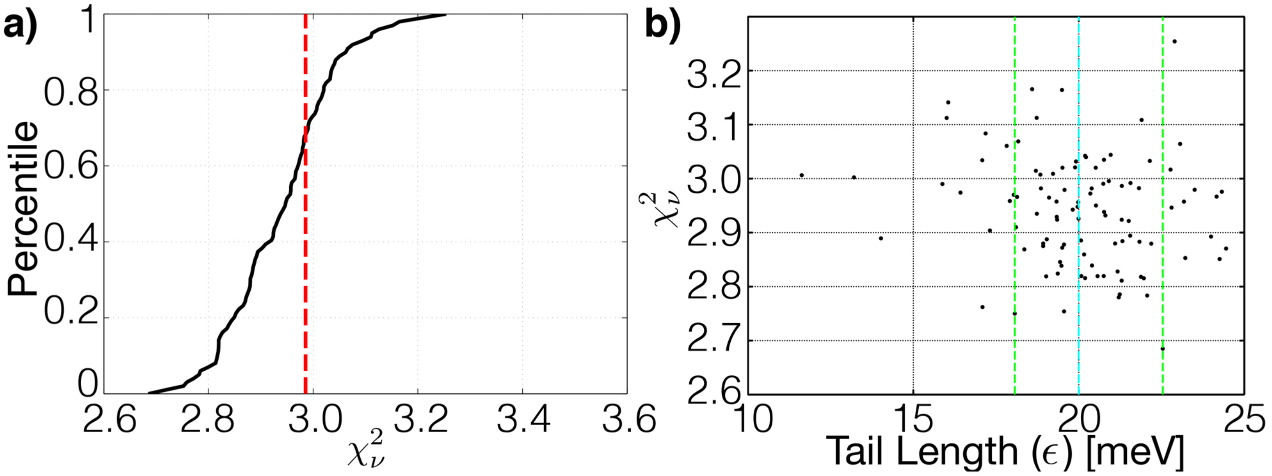

Figure S2. **a)** χ^2_ν cumulative distribution for PL measured at 123 K. The dashed red line denotes the 68th percentile, which indicates the threshold χ^2_ν value that sets the 1σ confidence interval for the joint probability distribution of all variables. **b)** Distribution of trion tail length, ε, for the PL measured at 123 K. The cyan vertical line denotes the best-fit value to the data, and the green vertical lines denote the upper and lower cut offs which contain 68% of the distribution, establishing the 1σ confidence interval for the single variable probability distribution for ε.

parameters: amplitude, position and width of all three peaks, the trion tail length, and the inhomogeneous broadening parameter. This is only one additional parameter, the trion tail length, relative to the number of parameters that are typically used, and all the parameters are well motivated by the physics. A small trapped exciton peak[9,10] was observed in the spectra below 198 K, which we were easily able to subtract from the spectra due to the large energy difference between the trapped state and the trion (see Supplementary Information S1).

The addition of the trion tail length to the set of typical fit parameters does complicate the fit procedure because the tail length and peak widths can compensate for each other creating unphysical, local χ^2 minima. We use a multi-step fit procedure to steer the fitting parameters away from these spurious, local minima. First, we take advantage of the separation between the trapped exciton peak and trion peak to fit the trapped exciton peak and background parameters independently[11]. These parameters are then held constant while performing an initial fit to the trion and exciton peaks. For some spectra, the peak widths and inhomogeneous broadening extracted at this stage are unphysically small, in which case we replace them with realistic values which are held constant during a refitting of the remaining parameters. Then a final fit is performed in which all parameters are free to minimize χ^2, yielding the best-fit overall parameters. This procedure was successful at yielding physical fit parameters for all but one spectrum, measured at 148 K, so we have removed this spectrum from all further analysis. Finally, we quantify the confidence intervals of our fit parameters by bootstrapping our data to perform a Monte Carlo simulation of our experiment[12]. Bootstrapping also estimates the distribution of each fit parameter for each spectrum. In all cases, the distribution contained a single peak clustered around the best-fit value, indicating the robustness of our approach (see below).

Fit errors were determined by bootstrapping the data to create 100 new data sets for each measured spectra. Each of the 100 new data sets was then fit to create a distribution of fit parameters with different χ^2 values. Confidence intervals were calculated by ranking fit results by their χ^2 values and increasing the cutoff χ^2 value until 68% of the fits or more fell between the upper and lower bounds set by the cutoff. Note that this was necessary as the Jacobian fit matrix was very flat at the bottom of the χ^2 potential, so simple first order propagation of error yielded erroneously large fit errors.

Fig. S2a shows the cumulative distribution of reduced chi squared, χ^2_ν, for 100 fits to the bootstrapped data from the PL measurements made at 123 K. The distribution appears like an error function suggesting that 100 samples is a good representative sample. Fig. S2b shows the distribution of trion tail lengths. The best-fit to the original data is shown in cyan, which the distribution is centered on. That there is no other value of the tail length around which the distribution is clustered is indicative of a robust fit. The green bars indicate the low and high cutoffs that surround 68% of the distribution and set the 1σ confidence interval for the trion tail length for the data measured at 123 K. All parameters for each of the PL measurements similarly show distributions clustered around a single value of the fit parameter, indicating a robust fit.

S6 Peak Red-Shift Temperature Dependence

The PL spectrum of a trion is the convolution of a Lorentzian with equation 1a in the main text

$$I_{tr}(E) = \frac{\Gamma}{\pi\varepsilon}\int_{-\infty}^{\infty} \mathrm{d}\tilde{E}\,\frac{\exp\left[-\left(E_{tr}^0-\tilde{E}\right)/\varepsilon\right]}{\left(E-\tilde{E}\right)^2+\Gamma^2}\Theta\left(E_{tr}^0-E\right) \tag{S6a}$$

$$= \frac{\Gamma}{\pi\varepsilon^2}\int_0^{\infty}\frac{e^{-x}\mathrm{d}x}{\left(x-\frac{E_{tr}^0-E}{\varepsilon}\right)^2+\left(\frac{\Gamma}{\varepsilon}\right)^2} = \frac{\Gamma}{\pi\varepsilon^2}\int_0^{\infty}\frac{e^{-x}\mathrm{d}x}{(x-\mathbb{X})^2+\gamma^2} \tag{S6b}$$

$$= \frac{\Gamma}{\pi\varepsilon^2}\frac{1}{\mathbb{X}^2+\gamma^2}\int_0^{\infty}\frac{e^{-x}\mathrm{d}x}{1-\frac{2\mathbb{X}x-x^2}{\mathbb{X}^2+\gamma^2}} = \frac{\Gamma}{\pi\varepsilon^2}\frac{1}{\mathbb{X}^2+\gamma^2}\int_0^{\infty}\mathrm{d}x\,e^{-x}\left[1+\frac{2\mathbb{X}x}{\mathbb{X}^2+\gamma^2}+\mathscr{O}\left(x^2\right)\right] \tag{S6c}$$

$$= \frac{\Gamma}{\pi}\frac{1}{\left(E_{tr}^0-E\right)^2+\Gamma^2}\left[1+\frac{2\varepsilon\left(E_{tr}^0-E\right)}{\left(E_{tr}^0-E\right)^2+\Gamma^2}+\cdots\right] \tag{S6d}$$

where in the second line we temporarily defined $\mathbb{X}$ as $\left(E_{tr}^0-E\right)/\varepsilon$ and γ as Γ/ε, and in the third line we have Taylor expanded the non-exponential part of the integrand about zero, a standard method for approximating integrals with exponential factors[13]. The peak position is where the derivative with respect to energy is zero, which gives the following implicit equation for the peak position as a function of temperature

$$\frac{\partial I_{tr}}{\partial E} = \frac{\Gamma}{\pi}\left\{\frac{2\left(E_{tr}^0-E-\varepsilon\right)}{\left[\left(E_{tr}^0-E\right)^2+\Gamma^2\right]^2}+\frac{8\varepsilon\left(E_{tr}^0-E\right)^2}{\left[\left(E_{tr}^0-E\right)^2+\Gamma^2\right]^3}+\cdots\right\} \tag{S7a}$$

$$\Rightarrow 0 = \left[\left(E_{tr}^0-E\right)^2+\Gamma^2\right]\left(E_{tr}^0-E-\varepsilon\right)+4\varepsilon\left(E_{tr}^0-E\right)^2+\cdots. \tag{S7b}$$

The temperature dependence is hidden in this equation in E_{tr}^0, ε, and Γ. However, we are not interested in the complete shift, as much of the shift will be due to band gap and binding energy renormalization, but the shift relative to E_{tr}^0. For convenience we will denote the shift we are interested in as $\Delta E = E_{tr}^0 - E$. Keeping only the terms we have explicitly calculated this gives a third order polynomial equation for ΔE, which we can Taylor expand in temperature about some temperature T_0.

$$0 = \Delta E^3 + 3\varepsilon\Delta E^2 + \Gamma^2\Delta E - \Gamma^2\varepsilon \tag{S8a}$$

$$0 = \left(3\Delta E^2+6\varepsilon\Delta E+\Gamma^2\right)\Delta E' + \left(3\Delta E^2-\Gamma^2\right)\varepsilon' + 2\Gamma(\Delta E-\varepsilon)\Gamma' \quad \text{(Derivative with respect to } T.\text{)} \tag{S8b}$$

$$\Rightarrow \Delta E(T) = \Delta E|_{T_0} + \left.\frac{\left(3\Delta E^2-\Gamma^2\right)\varepsilon'+2\Gamma(\Delta E-\varepsilon)\Gamma'}{3\Delta E^2+6\varepsilon\Delta E+\Gamma^2}\right|_{T_0}(T-T_0)+\cdots \tag{S8c}$$

where primes denote derivatives with respect to temperature. We could attempt to gain new information from this formula by using it to compare our peak fit parameters to our linear fit to peak shift versus temperature. However, the degree of approximation in the calculation is large and any new insights gained would be very speculative. Further, this formula is too complicated to gain physical intuition from, so we find it doesn't assist in understanding the data.

S7 Tail Length Low Temperature Expansion

The first three terms of the low temperature expansion of the tail length, equation (1b) from the main text, are

$$\varepsilon = \frac{m_X}{m_e}k_BT\left[1-\frac{m_X}{m_{tr}}\frac{4m_Xa^2}{\hbar^2}k_BT+\left(\frac{m_X}{m_{tr}}\frac{4m_Xa^2}{\hbar^2}k_BT\right)^2-\cdots\right]. \tag{S9}$$

This expansion shows that the temperature scale that controls the convergence of this series is

$$T_c = \frac{1}{k_B}\frac{m_{tr}}{m_X}\frac{\hbar^2}{4m_Xa^2} = \frac{409\,K\,nm^2}{a^2}. \tag{S10}$$

Values of T_c for the values of a used in Fig. 4c of the main text are shown in Table S2. Since T_c is lower when a is larger, higher order terms need to be included when a is larger, which explains the increasingly non-linear behavior observed for larger values of a. T_c is on the order of a few hundred Kelvin for most values of a in Fig. 4c, which necessitates the use of higher order terms to describe the tail length curves at even the lowest temperature, $< 83\,K$, in Fig. 4c.

a [nm]	0.54	0.96	1.35	1.78
T_c [K]	1403	444	224	129

Table S2. Convergence temperature scale for each value of a used in Fig. 4c of the main text.

References

1. Nie, Z. *et al.* Ultrafast carrier thermalization and cooling dynamics in few-layer MoS2. *ACS Nano* **8**, 10931–10940 (2014).
2. Lui, C. H. *et al.* Trion-induced negative photoconductivity in monolayer MoS2. *Phys. Rev. Lett.* **113**, 1–5 (2014).
3. Esser, a., Runge, E., Zimmermann, R. & Langbein, W. Photoluminescence and radiative lifetime of trions in GaAs quantum wells. *Phys. Rev. B - Condens. Matter Mater. Phys.* **62**, 8232–8239 (2000).
4. Zhang, C., Wang, H., Chan, W., Manolatou, C. & Rana, F. Absorption of light by excitons and trions in monolayers of metal dichalcogenide Mo S2: Experiments and theory. *Phys. Rev. B - Condens. Matter Mater. Phys.* **89**, 205436 (2014).
5. Berkelbach, T. C., Hybertsen, M. S. & Reichman, D. R. Theory of neutral and charged excitons in monolayer transition metal dichalcogenides. *Phys. Rev. B - Condens. Matter Mater. Phys.* **88**, 1–6 (2013).
6. Mak, K. F. *et al.* Tightly bound trions in monolayer MoS2. *Nat. Mater.* **12**, 207–211 (2012).
7. Nan, H. *et al.* Strong photoluminescence enhancement of MoS2 through defect engineering and oxygen bonding. *ACS Nano* **8**, 5738–5745 (2014).
8. Buscema, M., Steele, G. a., van der Zant, H. S. J. & Castellanos-Gomez, A. The effect of the substrate on the Raman and photoluminescence emission of single-layer MoS2. *Nano Res.* **7**, 1–11 (2014).
9. Mak, K. F., He, K., Shan, J. & Heinz, T. F. Control of valley polarization in monolayer MoS2 by optical helicity. *Nat. Nanotechnol.* **7**, 494–498 (2012).
10. Lagarde, D. *et al.* Carrier and polarization dynamics in monolayer MoS 2. *Phys. Rev. Lett.* **112**, 1–5 (2014).
11. Bevington, P. & Robinson, D. *Data Reduction and Error Analysis for the Physical Sciences* (McGraw-Hill, New York, 2003), third edit edn.
12. Press, W. H., Teukolsky, S. A., Vetterling, W. T. & Flannery, B. P. *Numerical Recipes, Third Edition* (Cambridge University press, New York, 2007).
13. Bender, C. M. & Orszag, S. A. *Advanced Mathematical Methods for Scientists and Engineers I* (Springer-Verlag, New York, 1999).
